# Supplementary material for: Enhancement of erythropoietic output by Cas9-mediated insertion of a natural variant in haematopoietic stem and progenitor cells
Source: Nat Biomed Eng. 2024 Jun 17;8(12):1540–52. doi: 10.1038/s41551-024-01222-6 (PMC11668683; doi:10.1038/s41551-024-01222-6)
Supplement: Supplementary file 1 — Supplementary figures. [file 41551_2024_1222_MOESM1_ESM.pdf]

nature biomedical engineering

Supplementary information

https://doi.org/10.1038/s41551-024-01222-6

Enhancement of erythropoietic output by Cas9-mediated insertion of a natural variant in haematopoietic stem and progenitor cells

In the format provided by the authors and unedited

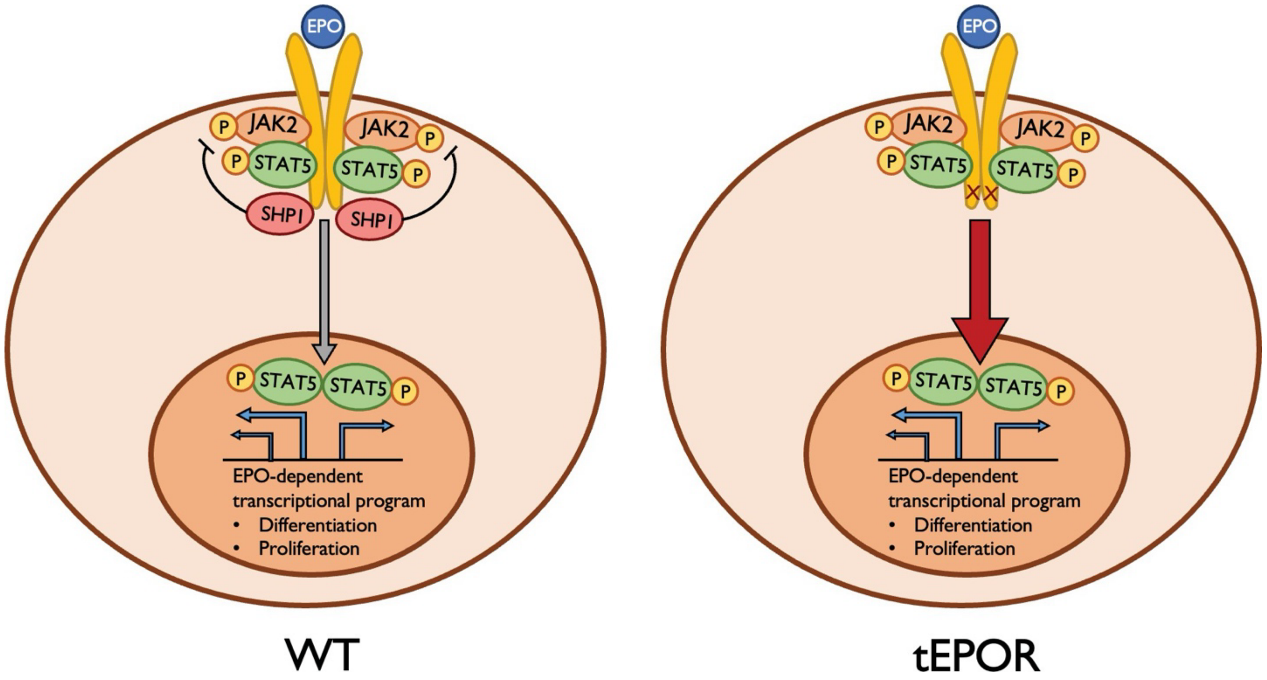

Supplementary Fig. 1 | Schematic of wild-type (WT) and tEPOR signaling cascade.
a, Visual representation of EPOR signaling cascade. SHP1 binds to intracellular inhibitory domain in WT cells, downregulating EPOR signaling. This domain is truncated in tEPOR cells making them hypersensitive to EPO.

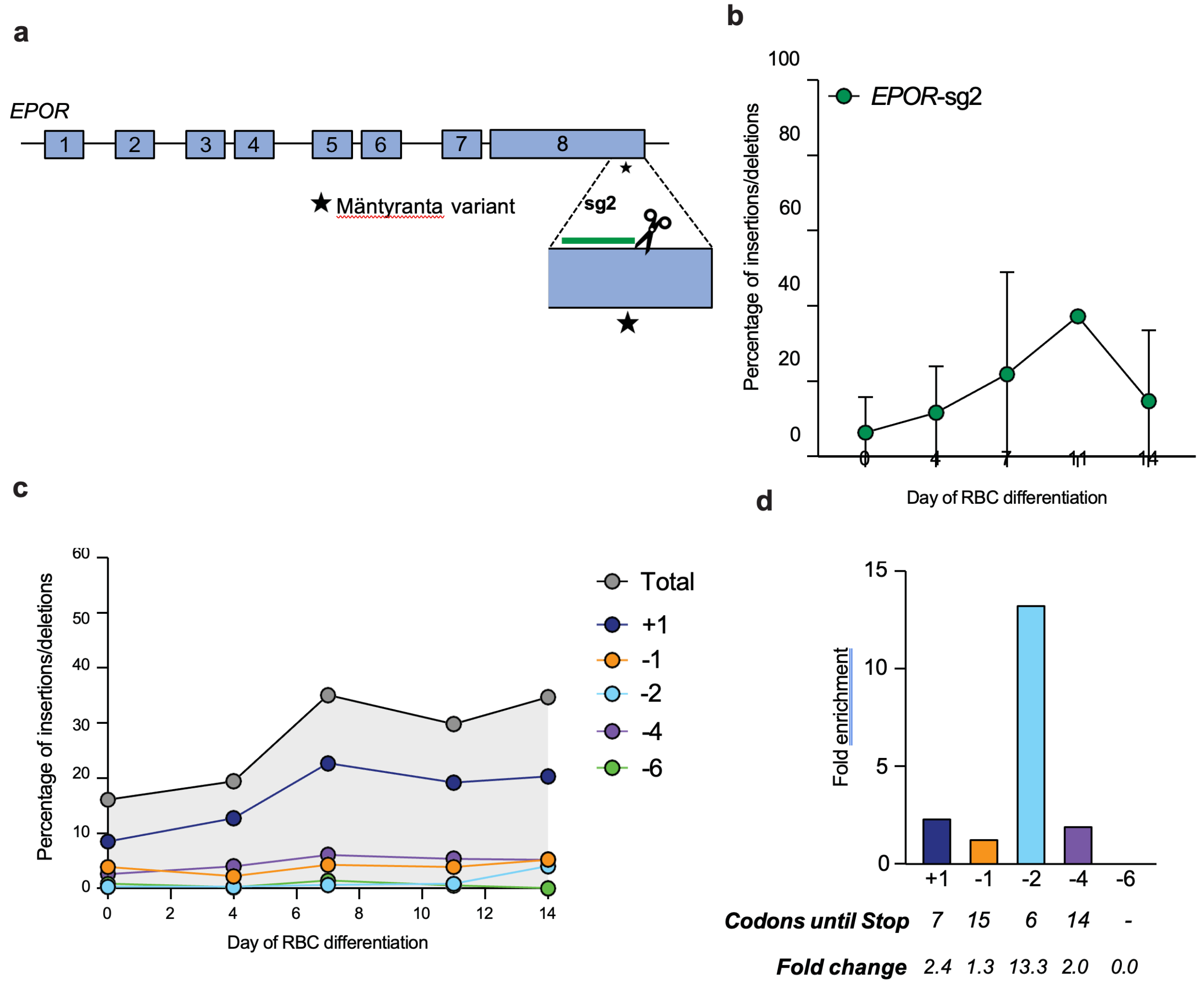

Supplementary Fig. 2 I Indel analysis of HSPCs edited with second candidate *EPOR* sgRNA (sg2) over course of erythroid differentiation. a, Schematic of *EPOR* gene and location of the second candidate sgRNA (*EPOR*-sg2) indicated by a line. Location of c.1316G>A mutation is denoted by the star **b,** Frequency of indels created by *EPOR*-sg2 in primary human CD34+ HSPCs over the course of erythroid differentiation. Points represent median ± interquartile range. Values represent N=4 biologically independent HSPC donors. **c,** Frequency of five most common indels found in one HSPC donor targeted with *EPOR*-sg2 over the course of RBC differentiation. **d,** Fold enrichment of five most common indels over course of RBC differentiation in one HSPC donor targeted with *EPOR*-sg2.

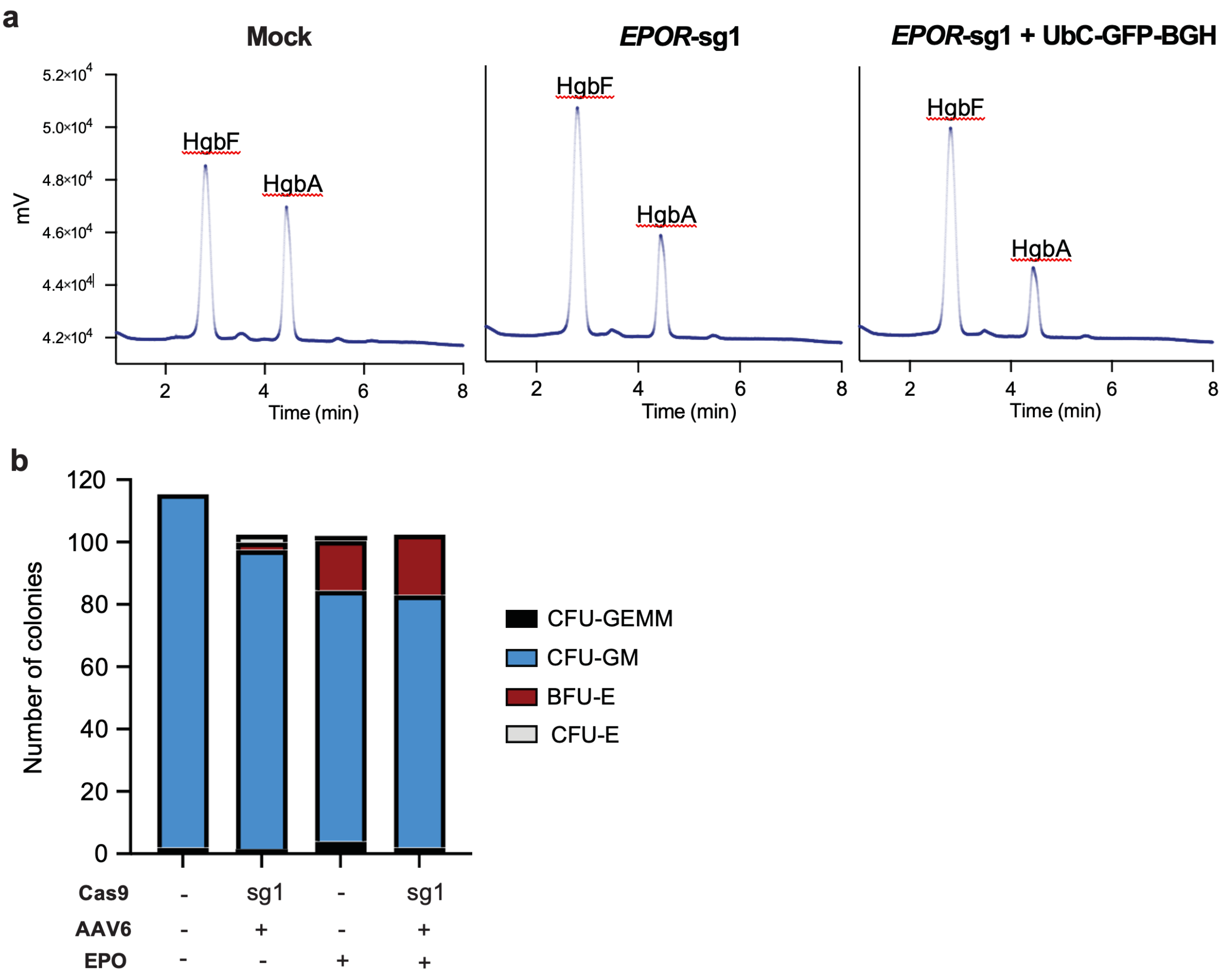

Supplementary Fig. 3 l HSPCs edited at *EPOR* locus show normal hemoglobin tetramers and maintain lineage formation. a, Representative HPLC plots of cells targeted with *EPOR*-sg1 at day 14 of RBC differentiation. HgbF=fetal hemoglobin, HgbA=adult hemoglobin. **b,** Colony formatting unit (CFU) assay of mock edited cells versus cells edited with *EPOR*-sg1 + BGH*.* Bars represent total number of colonies of each type: CFU-GEMM (multi-potential granulocyte, erythroid, macrophage, megakaryocyte progenitor cells), CFU-GM (colony forming unit-granulocytes and monocytes), BFU-E (erythroid burst forming units), CFU-E (colony forming unit-erythroid) colonies. N=1.

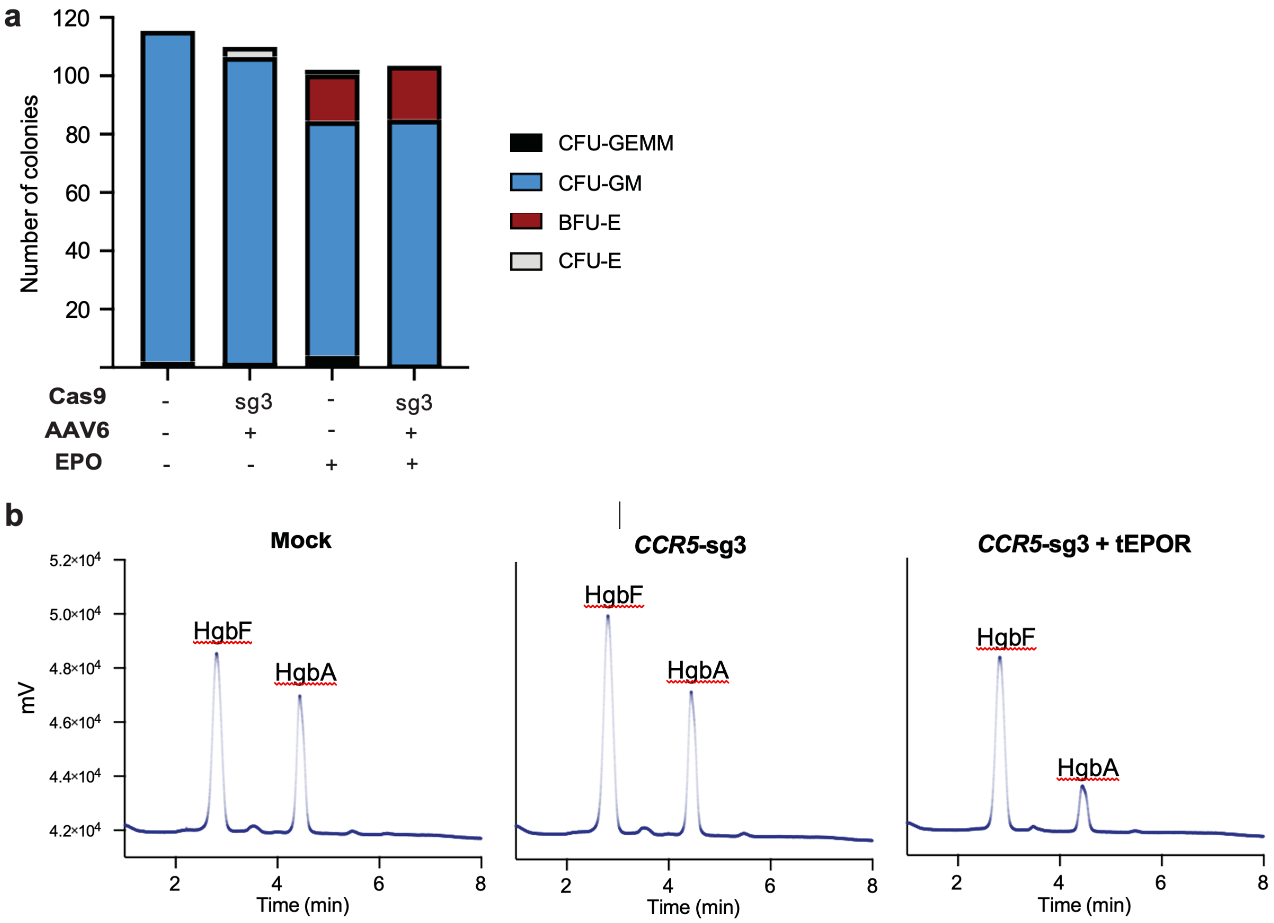

Supplementary Fig. 4 | Safe harbor integration of *tEPOR* does not affect HSPC lineage or hemoglobin formation. a, CFU assay of mock edited cells versus cells edited with *CCR5*-sg3 + *tEPOR.* Bars represent total number of colonies of each type: CFU-GEMM (multi-potential granulocyte, erythroid, macrophage, megakaryocyte progenitor cells), CFU-GM (colony forming unit-granulocytes and monocytes), BFU-E (erythroid burst forming units), CFU-E (colony forming unit-erythroid) colonies. N=1. Mock condition from Supplementary Fig. 3b shown here for comparison. **b,** Representative HPLC plots of cells targeted with *CCR5*-sg3 or *CCR5*-sg3 + *tEPOR* at day 14 of RBC differentiation. HgbF=fetal hemoglobin, HgbA=adult hemoglobin.

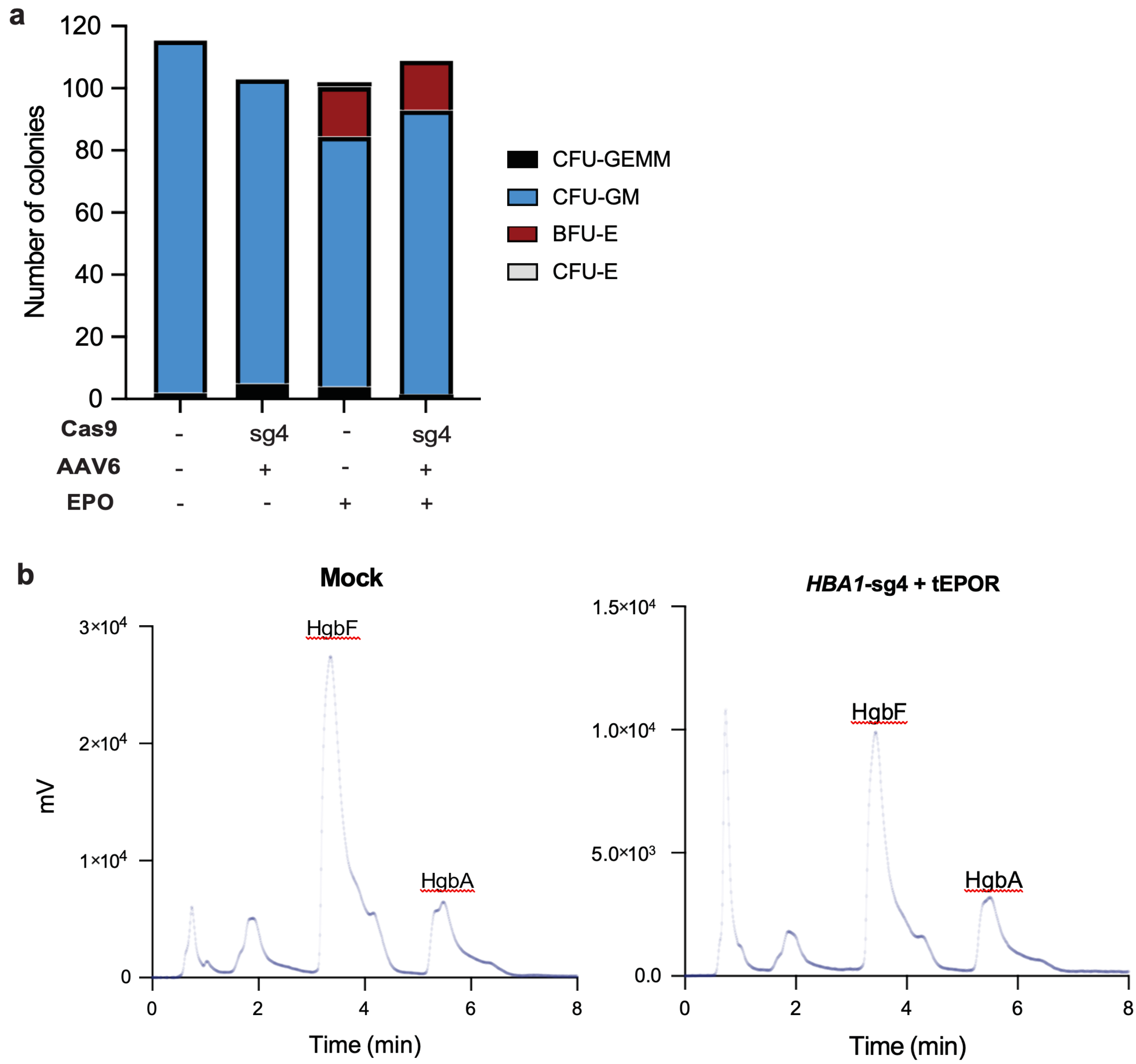

Supplementary Fig. 5 I Erythroid specific expression of *tEPOR* does not affect HSPC lineage or hemoglobin formation. a, CFU assay of mock edited cells versus cells edited with *HBA1*-sg4 + *tEPOR.* Bars represent total number of colonies of each type: CFU-GEMM (multi-potential granulocyte, erythroid, macrophage, megakaryocyte progenitor cells), CFU-GM (colony forming unit-granulocytes and monocytes), BFU-E (erythroid burst forming units), CFU-E (colony forming unit-erythroid) colonies. N=1. Mock condition from Supplementary Fig. 3b shown here for comparison. **b,** Representative HPLC plots of cells targeted with *HBA1*-sg4 + *tEPOR* at day 14 of RBC differentiation. HgbF=fetal hemoglobin, HgbA=adult hemoglobin.

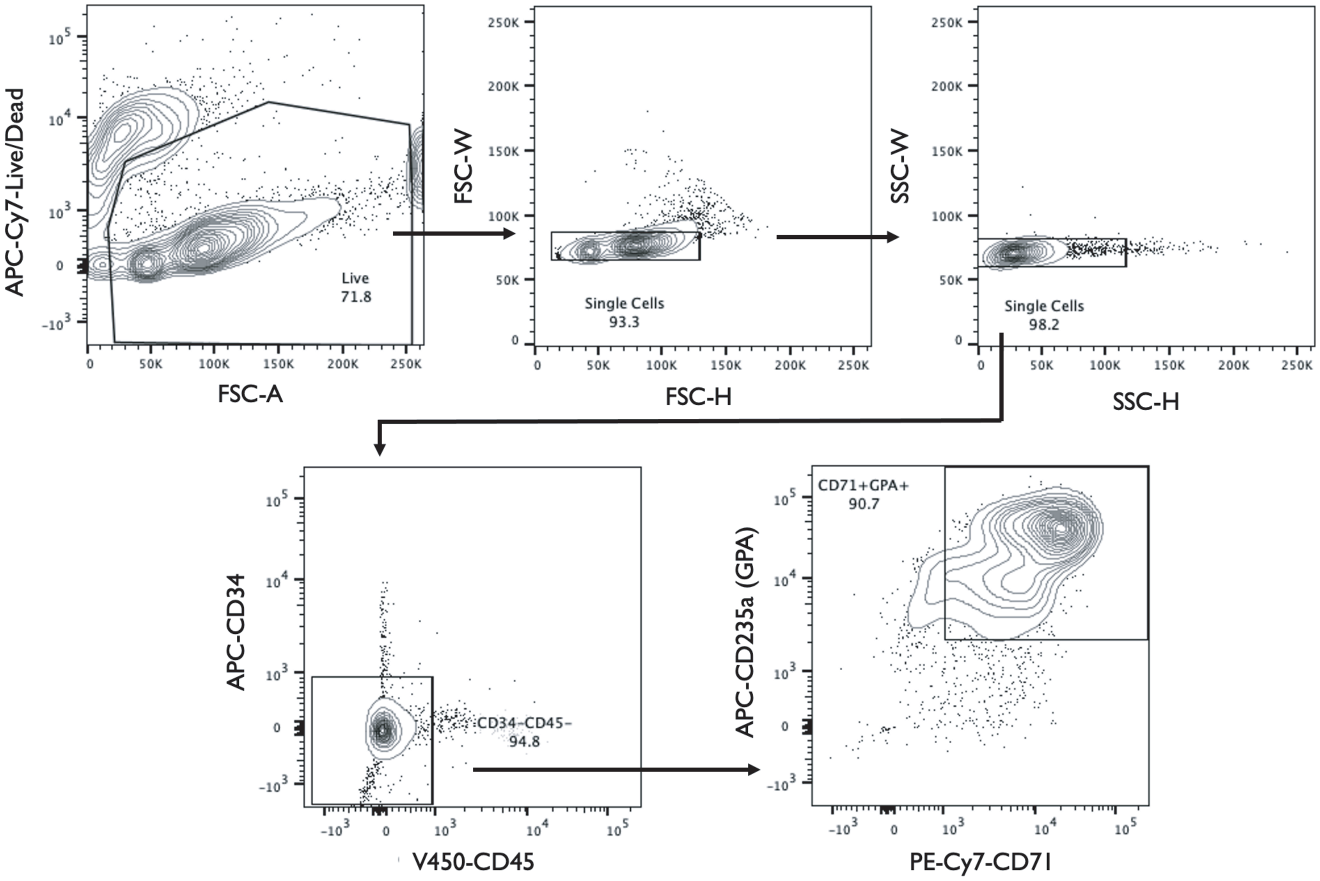

Supplementary Fig. 6 | Gating strategy for flow cytometry of RBC differentiation.
a, Representative flow plots of the gating strategy used to quantify RBC differentiation.
