# Supplementary material for: Enhancement of erythropoietic output by Cas9-mediated insertion of a natural variant in haematopoietic stem and progenitor cells
Source: Nat Biomed Eng. 2024 Jun 17;8(12):1540–52. doi: 10.1038/s41551-024-01222-6 (PMC11668683; doi:10.1038/s41551-024-01222-6)
Supplement: Supplementary file 2 — Reporting Summary [file 41551_2024_1222_MOESM2_ESM.pdf]

nature portfolio

Corresponding author(s): M. Kyle Cromer, Matthew Porteus

Last updated by author(s): April 22, 2024

Reporting Summary

Nature Portfolio wishes to improve the reproducibility of the work that we publish. This form provides structure for consistency and transparency in reporting. For further information on Nature Portfolio policies, see our Editorial Policies and the Editorial Policy Checklist.

Statistics

For all statistical analyses, confirm that the following items are present in the figure legend, table legend, main text, or Methods section.

n/a	Confirmed	
☐	☒	The exact sample size (*n*) for each experimental group/condition, given as a discrete number and unit of measurement
☐	☒	A statement on whether measurements were taken from distinct samples or whether the same sample was measured repeatedly
☐	☒	The statistical test(s) used AND whether they are one- or two-sided<br>*Only common tests should be described solely by name; describe more complex techniques in the Methods section.*
☒	☐	A description of all covariates tested
☒	☐	A description of any assumptions or corrections, such as tests of normality and adjustment for multiple comparisons
☐	☒	A full description of the statistical parameters including central tendency (e.g. means) or other basic estimates (e.g. regression coefficient) AND variation (e.g. standard deviation) or associated estimates of uncertainty (e.g. confidence intervals)
☐	☒	For null hypothesis testing, the test statistic (e.g. *F*, *t*, *r*) with confidence intervals, effect sizes, degrees of freedom and *P* value noted<br>*Give P values as exact values whenever suitable.*
☒	☐	For Bayesian analysis, information on the choice of priors and Markov chain Monte Carlo settings
☒	☐	For hierarchical and complex designs, identification of the appropriate level for tests and full reporting of outcomes
☒	☐	Estimates of effect sizes (e.g. Cohen's *d*, Pearson's *r*), indicating how they were calculated

Our web collection on statistics for biologists contains articles on many of the points above.

Software and code

Policy information about availability of computer code

Data collection	Microsoft Excel for Mac (v16.75.2) was used for data collection. ddPCR data were collected using QuantaSoft software (v1.7.4.0917, BioRad). Flow cytometry data were collected through FACS Diva 8 for FACS Aria II. STEMvision automated counter (STEMCELL Technologies) with manual correction was used to to determine the number of BFU-E, CFU-M, CFU-GM and CFU-GEMM colonies following the CFU assay.
Data analysis	GraphPad Prism 9 and Microsoft Excel for Mac (v16.75.2) were used for data analysis. INDEL frequency analysis was done through the TIDE analysis online tool (https://tide.nki.nl) on Sanger sequencing samples. FACS data were analysed through FlowJo 10.9.0 for Mac. For off-target analysis, FastQC (v0.11.8, http://www.bioinformatics.babraham.ac.uk/projects/fastqc, default parameters) was employed to assess the quality of raw reads. Subsequently, paired-end reads were aligned to the specified off-target regions using CRISPResso2 (version 2.2.14, CRISPResso --fastq_r1 reads_r1.fastq.gz --fastq_r2 reads_r2.fastq.gz --amplicon_seq).

For manuscripts utilizing custom algorithms or software that are central to the research but not yet described in published literature, software must be made available to editors and reviewers. We strongly encourage code deposition in a community repository (e.g. GitHub). See the Nature Portfolio guidelines for submitting code & software for further information.

Data

Policy information about availability of data

All manuscripts must include a data availability statement. This statement should provide the following information, where applicable:

- Accession codes, unique identifiers, or web links for publicly available datasets
- A description of any restrictions on data availability
- For clinical datasets or third party data, please ensure that the statement adheres to our policy

The main data supporting the results in this study are available within the paper and its Supplementary Information. High-throughput-sequencing data generated for off-target analysis is available through the NCBI Sequence Read Archive database via the accession number PRJNA1102034. Sequences of the guide RNA and ddPCR primers and probes are provided in Methods. Source data for the figures are provided with this paper. All data generated in this study are available from the corresponding authors on reasonable request.

Research involving human participants, their data, or biological material

Policy information about studies with human participants or human data. See also policy information about sex, gender (identity/presentation), and sexual orientation and race, ethnicity and racism.

Reporting on sex and gender	Primary cell samples used in this study were de-identified.
Reporting on race, ethnicity, or other socially relevant groupings	Primary cell samples used in this study were de-identified.
Population characteristics	Primary cell samples used in this study were de-identified.
Recruitment	Primary cord-blood and peripheral-blood CD34+ HSPCs were derived from healthy donors at random. HSPCs from patients with sickle cell disease were provided by Vivien A. Sheehan of Emory University (formerly at Baylor College of Medicine).
Ethics oversight	The Stanford IRB committee approved the research on healthy donor HSPCs under protocol #33813. Cells from patients with sickle cell disease were collected under the Baylor College of Medicine IRB protocol H-41213.

Note that full information on the approval of the study protocol must also be provided in the manuscript.

Field-specific reporting

Please select the one below that is the best fit for your research. If you are not sure, read the appropriate sections before making your selection.

☒ Life sciences ☐ Behavioural & social sciences ☐ Ecological, evolutionary & environmental sciences

For a reference copy of the document with all sections, see nature.com/documents/nr-reporting-summary-flat.pdf

Life sciences study design

All studies must disclose on these points even when the disclosure is negative.

Sample size	Experiments were performed in 2–3 biological HSPC donors.
Data exclusions	No data were excluded from the analyses.
Replication	All attempts at replication were successful.
Randomization	The samples were allocated at random.
Blinding	There was no blinding in this study.

Reporting for specific materials, systems and methods

We require information from authors about some types of materials, experimental systems and methods used in many studies. Here, indicate whether each material, system or method listed is relevant to your study. If you are not sure if a list item applies to your research, read the appropriate section before selecting a response.

Materials & experimental systems

n/a	Involved in the study	
☐	☒	Antibodies
☐	☒	Eukaryotic cell lines
☒	☐	Palaeontology and archaeology
☒	☐	Animals and other organisms
☒	☐	Clinical data
☒	☐	Dual use research of concern
☒	☐	Plants

Methods

n/a	Involved in the study	
☒	☐	ChIP-seq
☐	☒	Flow cytometry
☒	☐	MRI-based neuroimaging

Antibodies

Antibodies used	APC anti-CD34 (BioLegend, cat: 343510, Clone 581, Lot B377029), V450 anti-CD45 (BD Biosciences cat: 560367 Clone: HI30, Lot 1249199), PE-Cy7 anti-CD71 (eBioscience cat: 25071942, Clone: OKT9, Lot 2450617), PE anti-CD235a (eBioscience cat: 12998782, Clone: HIR2, Lot 2450518), Ghost Dye Red 780 (Tonbo Biosciences)
Validation	APC anti-CD34 antibody has been validated for flow cytometry in human peripheral blood leukocytes and cited in 18 publications according to the manufacturer's website. V450 anti-CD45 antibody has been validated for flow cytometry in human lymphocytes according to the manufacturer's website. PE-Cy7 anti-CD71 antibody has been validated for flow cytometry in human peripheral blood cells and cited in 23 publications according to the manufacturer's website. PE anti-CD235a antibody has been validated for flow cytometry in human peripheral blood cells and cited in 36 publications according to the manufacturer's website.

Eukaryotic cell lines

Policy information about cell lines and Sex and Gender in Research

Cell line source(s)	HEK-293T cells were acquired from ATCC.
Authentication	HEK-293T cells were authenticated by STR profiling at ATCC.
Mycoplasma contamination	The cell line was tested for mycoplasma by the manufacturer, and was found to be negative.
Commonly misidentified lines (See ICLAC register)	No commonly misidentified cell lines were used.

Flow Cytometry

Plots

Confirm that:

☒ The axis labels state the marker and fluorochrome used (e.g. CD4-FITC).

☒ The axis scales are clearly visible. Include numbers along axes only for bottom left plot of group (a 'group' is an analysis of identical markers).

☒ All plots are contour plots with outliers or pseudocolor plots.

☒ A numerical value for number of cells or percentage (with statistics) is provided.

Methodology

Sample preparation	Cells were washed once with FACS buffer and then incubated with 4-antibody cocktail (APC anti-CD34, V450 anti-CD45, PE-Cy7 anti-CD71 and PE anti-CD235a) for 15–30 minutes at 4 degrees Celsius. The cells were then washed with FACS buffer. Cells were resuspended in fresh buffer and stained with Ghost Dye Red 780 Live/Dead for at least 5 minutes before analysis.
Instrument	FACS Aria II cytometer (BD Biosciences).
Software	FACS Diva software 8 was used for data collection. FACS data analysis was performed using FlowJo (10.9.0 for Mac) software.
Cell population abundance	n/a
Gating strategy	Cells were first gated by FSC-A and Live/Dead. Doublet discrimination was performed using FSC-H/FSC-W and SSC-H/SSC-W. plots. CD34– and CD45– cells were then gated followed by CD71+/CD235a (GPA)+. GFP+ cells were gated by comparing to Mock-edited cells.

☒ Tick this box to confirm that a figure exemplifying the gating strategy is provided in the Supplementary Information.
